# Notes and Comments

**Published:** 1894-04

**Authors:** 


					﻿Notes and Comments.1
1 The assistant editor solicits contributions for this department,—new
methods, new remedies and formulas, or any short practical note which may
prove of value to the practitioner or student. Address 212 South Fifteenth
Street, Philadelphia.
The Micro-organisms of the Human Mouth.—We have re-
ceived a copy of an essay upon the relationship of micro-organisms
to dental caries. The address was delivered by Professor Albert
P. Brubaker before the Woman’s Dental Association; this subject
is one of much importance to dentistry, and the reading of the
paper—being illustrated with culture-tubes, microscopic prepara-
tions, diagrams, etc.—was of unusual interest.
Of course the credit of solving the problem of dental caries
belongs to Professor Miller, of Berlin, and it is a cause for con-
gratulation that we have among us such able teachers and men
capable of so much valuable and original work. It is also gratify-
ing that this line of investigation and demonstration is being carried
on by other members of our own household.
We congratulate the Woman’s Dental Association upon having
the subject so ably presented. It is desirable and essential for every
dental practitioner to make himself or herself familiar with the
study in order to achieve successful results in dental therapeutics
as measured by the advance standards.
				

